# Effects of Phenolic Compounds on Biofilm Formation by Table Olive‐Related Microorganisms

**DOI:** 10.1002/fsn3.4634

**Published:** 2024-12-01

**Authors:** Elio López‐García, Antonio Benítez‐Cabello, Francisco Noé Arroyo‐López

**Affiliations:** ^1^ Food Biotechnology Department, Instituto de la Grasa (CSIC) Campus Universitario Pablo de Olavide Seville Spain

**Keywords:** biofilm, hydroxytyrosol, *Lactiplantibacillus*, oleuropein, table olives, tyrosol, yeasts

## Abstract

The process of biofilm formation during table olive fermentation is crucial to turning this fermented vegetable into a probiotic food. Some phenolic compounds have been described as important quorum‐sensing molecules during biofilm development. The present in vitro study examined the effects of three phenolic compounds widely found in table olive fermentations (Oleuropein 0–3000 ppm, Hydroxytyrosol 0–3000 ppm, and Tyrosol 0–300 ppm) on the development of single biofilm by diverse microorganisms isolated from table olives (*Lactiplantibacillus pentosus* 13B4, Lp119, and LPG1; *Lactiplantibacillus plantarum* Lp15 and LAB23; and yeasts *Wickerhamomyces anomalus* Y12, *Candida boidinii* Y13, and *Saccharomyces cerevisiae* Y18). Biofilm formation was quantified in vitro by crystal violet staining in microtiter plates after incubation at 30°C for 96 h. A clear tendency to decrease the biofilm production was observed for the *L. plantarum* strains when any of the three phenolic compounds were added to the medium, which was statistically significant (*p* ≤ 0.05) for certain concentrations and phenols. In the case of yeasts, no statistical influence on biofilm formation was noticed when the phenolic compounds were dosed to the culture medium. Finally, the effects of the phenolic compounds on the *L. pentosus* strains were dependent on the strain assayed. Thereby, addition of phenolic compounds on 13B4 or Lp119 strains did not have statistical influence on biofilm production. On the contrary, the probiotic LPG1 strain noticed a statistical increase in biofilm production when a low concentration of tyrosol (50 ppm) was added to the medium. Results obtained in this work could be useful to control the biofilm formation process on olive epidermis during table olive fermentation to include beneficial microorganisms.

## Graphical abstract

This in vitro study investigated the effects of three phenolic compounds commonly found in table olive fermentations on biofilm formation by various microorganisms isolated from table olives. The findings revealed that while *Lactiplantibacillus plantarum* strains showed a significant reduction in biofilm production with certain phenolic concentrations, the *L. pentosus* strain LPG1 exhibited a notable increase in biofilm production with low tyrosol levels, highlighting the potential for controlling biofilm formation to promote beneficial microorganisms in table olive fermentation.

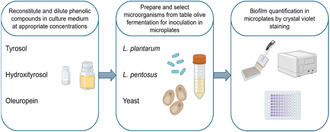

## 1 Introduction

Table olives are one of the most important fermented vegetables in Mediterranean countries, with a worldwide production of around 3 million tons/year (IOOC 2024). Microorganisms play an important role during table olive fermentation, determining the quality, safety, and flavor of the final product. Among the microbial species with a major importance in the fermentation of table olives, we can find *Lactiplantibacillus pentosus* and *Lactiplantibacillus plantarum* among lactic acid bacteria (LAB) (Hurtado et al. 2012), and *Saccharomyces cerevisiae, Wickerhamomyces anomalus*, and *Candida boidinii* among yeasts (Arroyo‐López, Romero‐Gil et al. 2012).

Fermented vegetables, especially table olives, are appropriate carriers of probiotic microorganisms (Peres et al. 2012). Recent research has highlighted the significant probiotic activities of microorganisms isolated from table olive fermentations (Benítez‐Cabello et al. 2019; Benítez‐Cabello et al. 2020; Coimbra‐Gomes et al. 2022; Guantario et al. 2018; López‐García, Benítez‐Cabello, Arenas‐de Larriva et al. 2023; Porru et al. 2018; Simões et al. 2021), and biofilms are critical to turn table olives into a carrier of these probiotic microorganisms for final consumers. Biofilms are defined as complex microbial communities often integrated by diverse species, adhered to biotic or abiotic surfaces, and embedded in an extracellular polymeric substance (exopolysaccharides) produced by themselves. These entities are the predominant mode of microbial growth. Recent studies have shown that these complex polymicrobial communities are formed by LAB and yeast species during table olive fermentations (Domínguez‐Manzano et al. 2012; Arroyo‐López, Bautista‐Gallego, et al. 2012; Grounta et al. 2016). These multispecies biofilms develop preferably on the surface of the olive epidermis, in contact with the brines in which they are submerged.

The reason for the formation of these structures in table olives is still unknown. One hypothesis is that the elevated concentration of nutrients (including amino acids, sugars, vitamins, etc.) at the brine/olive interface establishes a concentration gradient. Microorganisms can sense this gradient and are thereby induced to migrate from the brine to the olive surface. But biofilms are also forms of resistance, and they could protect microorganisms from the harsh environmental conditions found in the table olive environment such as high NaCl concentrations, low pH values, or the presence of antimicrobial compounds such as polyphenols.

Polyphenols represented 2%–3% of the olive flesh, which include flavonoids, phenolic acids, phenolic alcohols (here we include hydroxytyrosol and tyrosol), and secoiridoids (category for oleuropein) (Tekaya et al. 2022). Table olive processing decreases oleuropein levels with concomitant increases in the hydrolysis products hydroxytyrosol and tyrosol (Charoenprasert and Mitchell, 2012). This study aims to determine whether these changes could favor or inhibit the biofilm formation process and whether they affect table olive‐associated microorganisms uniformly. Certain phenolic compounds such as tyrosol have been identified as key quorum‐sensing molecules in yeasts, influencing biofilm formation or detachment (Rodrigues and Cernáková 2020); however, there is limited information regarding their effects on bacteria.

## 2 Material and Methods

### 2.1 Microorganisms

A total of 3 *L. pentosus* (Lp119, 13B4, and LPG1), 2 *L. plantarum* (Lp15 and LAB23), and 3 yeast (*W. anomalus* Y12, *C. boidinii* Y13, and *S. cerevisiae* Y18) strains, all of them previously isolated from table olive processing and identified by molecular methods, were used in the present study. These microorganisms belong to the Table Olive Microbial Collection (TOMC) of Instituto de la Grasa (CSIC, Seville, Spain), and they have been selected by their technological and probiotic features (Benítez‐Cabello et al. 2019, 2020; López‐García, Benítez‐Cabello, Arenas‐de Larriva et al. 2023; León‐Romero et al. 2016; Rodríguez‐Gómez et al. 2012).

### 2.2 Phenolic Compounds

The commercial phenolic compounds used in this work were oleuropein (Extrasynthase, France), hydroxytyrosol (Extrasynthase, France), and tyrosol (Extrasynthase, France), all with a purity > 98%. The lyophilized phenolic compound was reconstituted in sterile purified water and subsequently filtered using 0.22 μm filters. Following filtration, the compounds were diluted to achieve the required concentration ranges for the experiments. Table 1 shows the phenolic compounds and the concentration ranges assayed in this study.

**TABLE 1:** Table olive phenolic compounds and their concentrations added to synthetic medium.

Oleuropein, ppm (mg/L)	0	200	500	1000	2000	3000
Hydroxytyrosol, ppm (mg/L)	0	200	500	1000	2000	3000
Tyrosol, ppm (mg/L)	0	20	50	100	200	300

### 2.3 Culture Medium

The basal growth medium used in this study was Man, Rogosa, and Sharpe (MRS) broth for LAB (Oxoid, Basingstoke, Hampshire, UK) and yeast‐malt‐peptone‐glucose (YM) broth for yeasts (Difco, Becton Dickinson and Company, Sparks, MD, USA). Both media were filtered through 0.22 μm filters and modified by adding varying concentrations of three different phenolic compounds prior to inoculation.

### 2.4 Testing Biofilm Formation

The microbial growth was monitored using 96‐well microplates incubated for 96 h at 30°C with the SPECTROstar Nano plate reader (BMG Labtech, Ortenberg, Germany). The microplate wells were filled with 200 μL of MRS broth medium for bacteria or 200 μL of YM broth medium for yeast, both modified to different concentrations of each phenolic compound (Table 1), and then inoculated with 10 μL of the strain suspension, reaching an initial OD_595_ value above 0.1 (inoculum level of 6 log_10_ CFU/well). For each experimental condition, negative controls (uninoculated wells) were included to subtract the noise signal when determining biofilm formation. The complete set of experiments was replicated 5 times.

The microbial biofilm formation was determined using the protocol described by Toledo‐Arana et al. (2001) with slight modifications. Briefly, after the growth of the strains in 96‐well microplates, the supernatant was removed and the wells were washed twice with sterile PBS. Subsequently, 200 μL of a 0.8% aqueous crystal violet solution was added and incubated for 15 min at room temperature. Afterward, the crystal violet solution was removed from the wells and washed twice with sterile PBS. Finally, to extract the crystal violet from the cells adhered to the surface, 100 μL of an ethanol and acetone mixture (80:20) was used. The OD_595_ was then determined using the SPECTROstar Nano microplate reader.

### 2.5 Statistical Analysis

A factorial ANOVA analysis was executed with the Statistical 7.0 software package (StatSoft Inc., Tulsa, OK, USA), using “biofilm formation” as a dependent variable and “type of strain” (*n* = 7; Lp15, LAB23, Lp119, LPG1, 13B4, Y12, Y13, and Y18) and “phenols” (*n* = 4; control‐absence of phenols, tyrosol, hydroxytyrosol, and oleuropein) as categorical variables. To determine significant differences among treatments (*p ≤* 0.05), a post hoc Scheffé comparison test was carried out.

## 3 Results

In this study, in vitro biofilm formation was determined by crystal violet staining in a total of 720 wells (8 microbial strains × 3 olive phenolic compounds × 6 concentrations × 5 replicated) after 96 h of incubation at 30°C. Exogenous phenolic compounds were commercially obtained and added to the specific culture media in a range of concentrations usually found during table olive fermentations.

**FIGURE 1:**
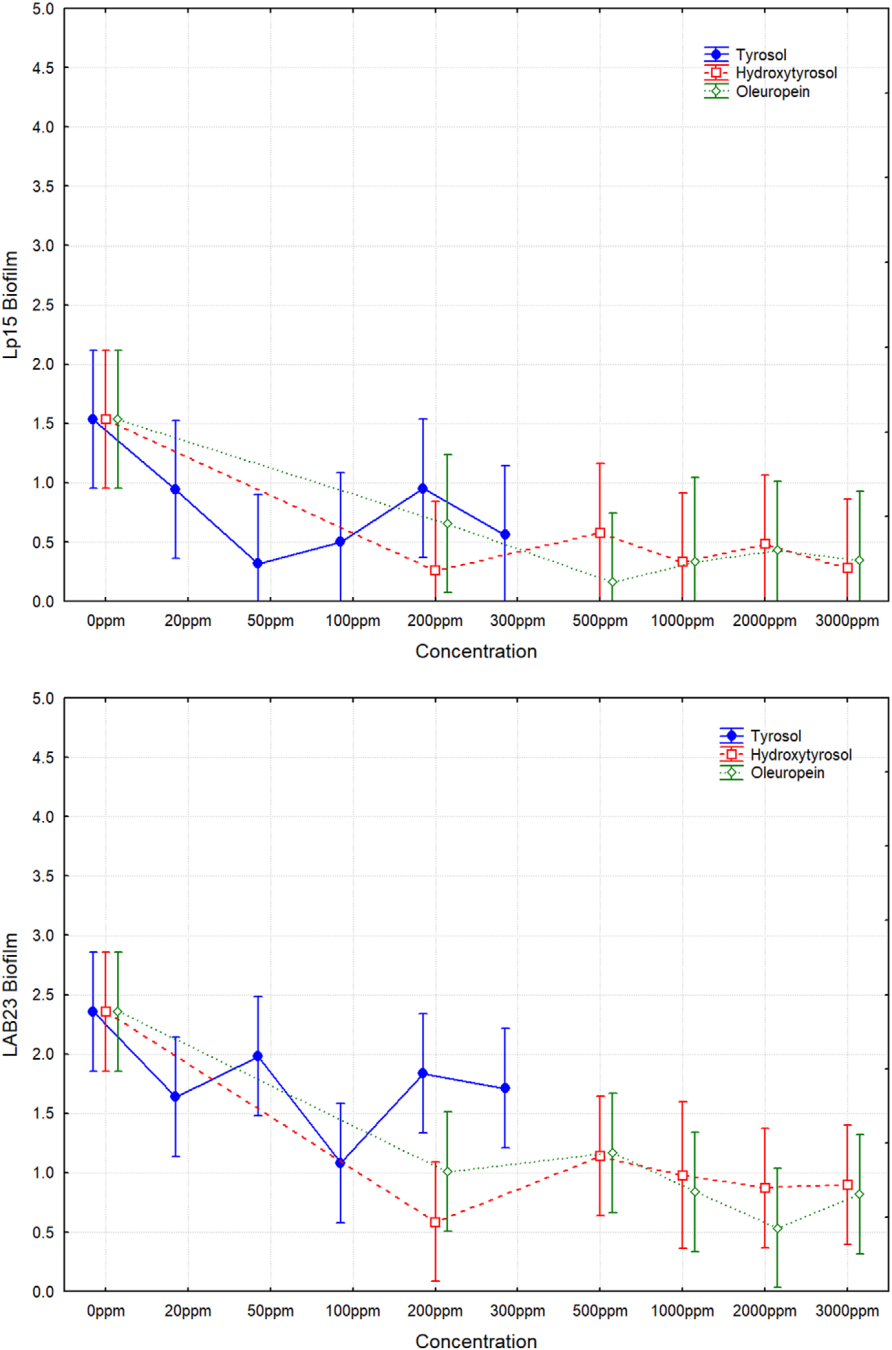
In vitro biofilm formation of *Lactiplantibacillus plantarum* Lp15 and LAB23 strains as a function of different concentrations of tyrosol (0–300 ppm), hydroxytyrosol (0–3000 ppm), and oleuropein (0–3000 ppm) added to culture medium. Means ± least squares. Vertical bars denote 0.95 confidence intervals.

Figure 1 shows the effects of the varying concentrations of phenols assayed on biofilm formation in the two *L. plantarum* strains (LAB23 and Lp15). Clearly, biofilm production decreased when table olive phenolic compounds were added to the culture medium. In the case of tyrosol, albeit there was a clear tendency to reduce the biofilm production for both *L. plantarum* strains, the differences were not statistically significant compared to the control treatment (0 ppm). However, for hydroxytyrosol and oleuropein, many of the concentrations tested above 200 ppm statistically reduced the biofilm production compared to the control treatment, which was especially evident for the LAB23 strain. In absence of phenols, LAB23 had a higher biofilm production (2.35 ± 0.53) than the Lp15 strain (1.56 ± 1.15), but without significant differences between them.

**FIGURE 2:**
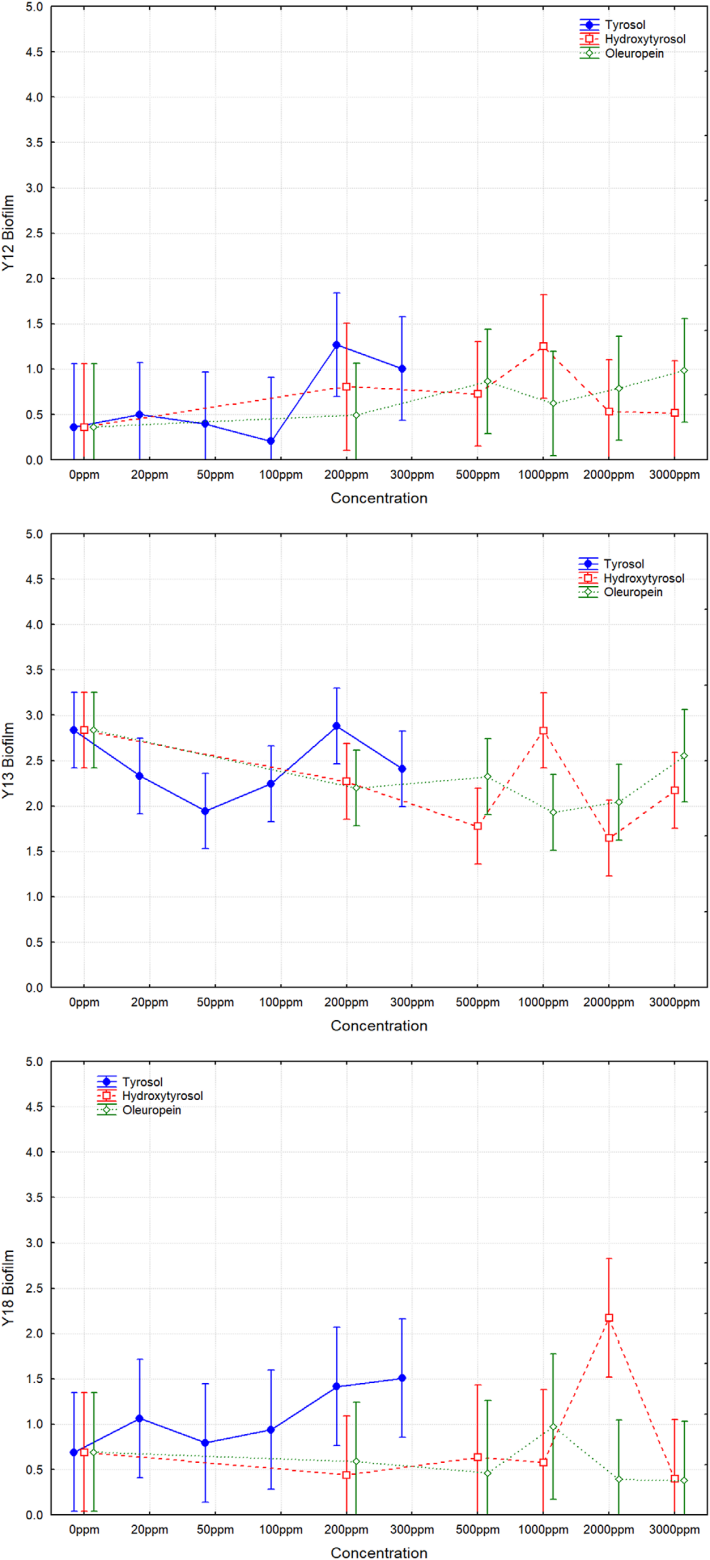
In vitro biofilm formation of yeasts *Wickerhamomyces anomalus* Y12, *Candida boidinii* Y13, and *Saccharomyces cerevisiae* Y13 as a function of different concentrations of tyrosol (0–300 ppm), hydroxytyrosol (0–3000 ppm), and oleuropein (0–3000 ppm) added to culture medium. Means ± least squares. Vertical bars denote 0.95 confidence intervals.

Figure 2 shows the effects of the different concentrations of phenols assayed on biofilm formation by the three yeast species isolated from table olive processing. Within the tested concentrations, the addition of phenols did not result in any statistically significant changes in the biofilm production compared to the absence of these compounds, except for *S. cerevisiae* Y18 at 2000 ppm hydroxytyrosol, where an increase in the biofilm production was noticed. In absence of phenols, *C. boidinii* Y13 had a statistically higher biofilm production (2.83 ± 0.30) than *W. anomalus* Y12 (0.35 ± 0.16) or *S. cerevisiae* Y18 (0.69 ± 0.13).

**FIGURE 3:**
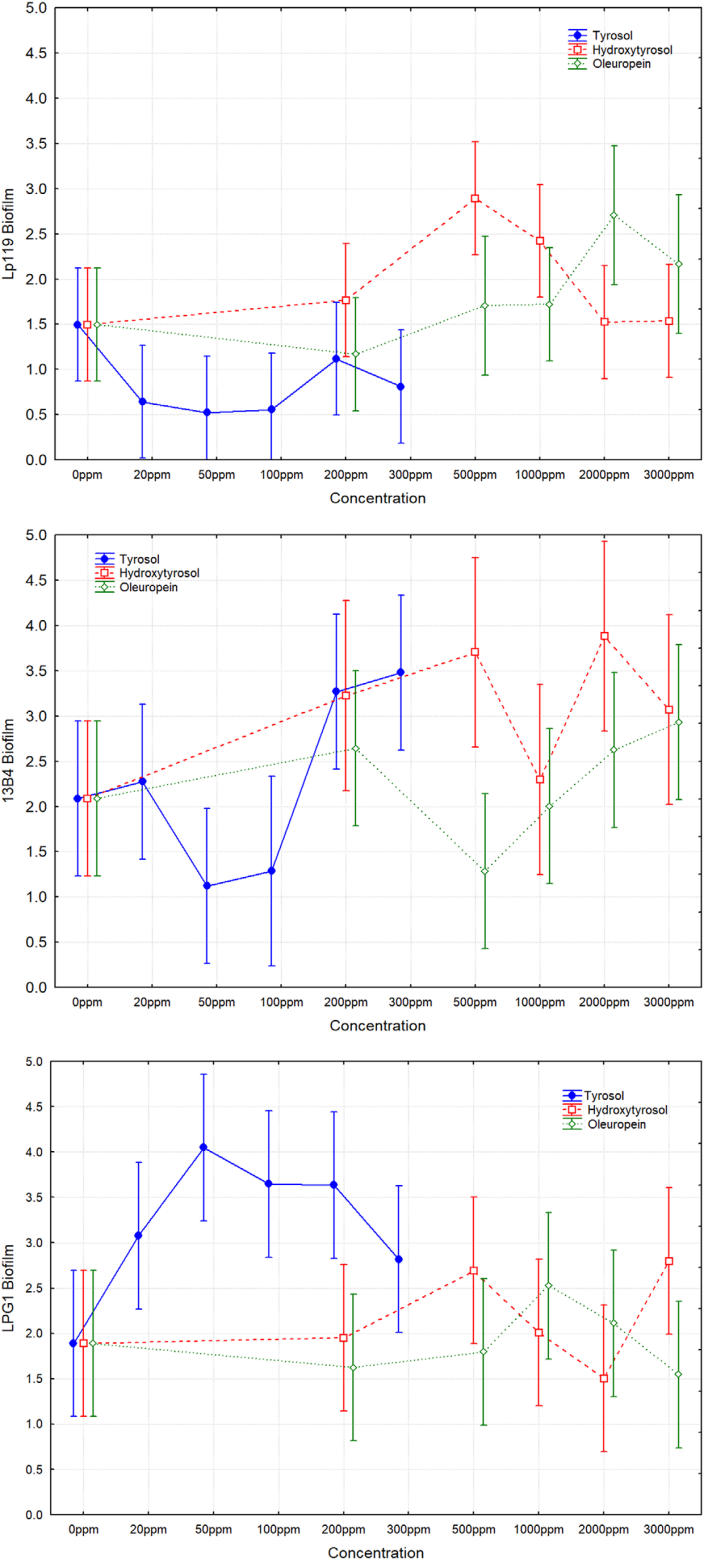
In vitro biofilm formation of *Lactiplantibacillus pentosus* Lp119, 13B4, and LPG1 strains as a function of different concentrations of tyrosol (0–300 ppm), hydroxytyrosol (0–3000 ppm), and oleuropein (0–3000 ppm) added to culture medium. Means ± least squares. Vertical bars denote 0.95 confidence intervals. Means ± least squares. Vertical bars denote 0.95 confidence intervals.

The behavior of the *L. pentosus* strains was different as a function of the type of phenols and concentration tested (Figure 3). In absence of phenols, there were not statistically significant differences for biofilm production among the three *L. pentosus* strains (Lp119 1.49 ± 0.24, 13B4 2.06 ± 0.49, and LPG1 1.88 ± 0.19). However, when 50 ppm of tyrosol was added to the medium, the probiotic LPG1 strain statistically increased its biofilm production, reaching a value of 4.02, which did not happen for the rest of the phenols or strains. The 13B4 strain also considerably increased its biofilm production when 2000 ppm of hydroxytyrosol (3.95) or 300 ppm of tyrosol (3.50) were added to the medium, but in this case there were not statistically significant differences compared to the control (absence of phenols).

**FIGURE 4:**
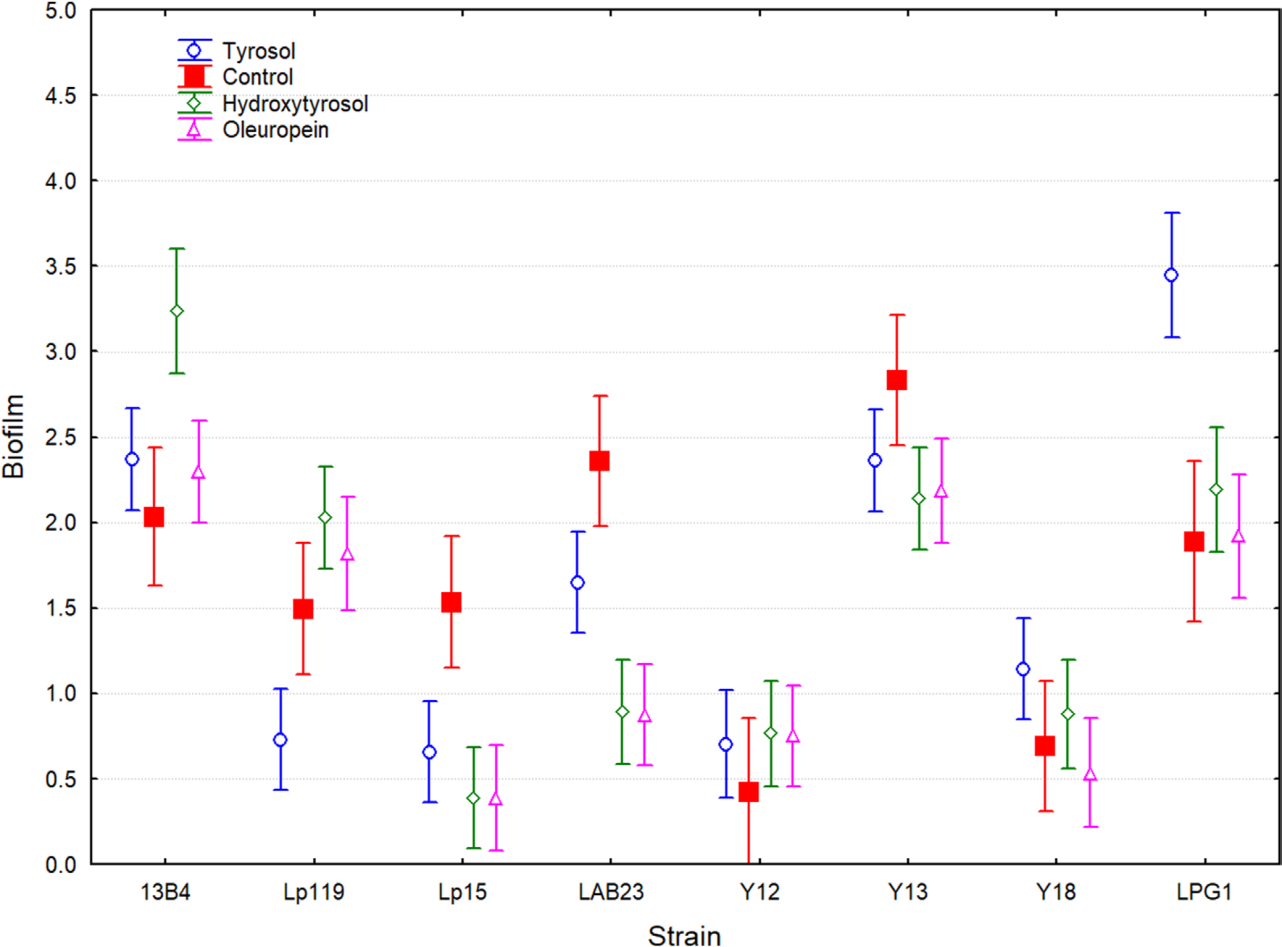
Factorial ANOVA analysis for the in vitro biofilm formation (dependent variable) as a function of categorical variables type of strain (*Lactiplantibacillus plantarum* Lp15 and LAB23; *L. pentosus* 13B4, Lp119, and LPG1; yeasts *Wickerhamomyces anomalus* Y12, *Candida boidinii* Y13, and *Saccharomyces cerevisiae* Y18) and type of phenols (control, absence of phenols; tyrosol; hydroxytyrosol; oleuropein). Means ± least squares. Vertical bars denote 0.95 confidence intervals.

Finally, a factorial ANOVA analysis was executed to determine statistical differences for biofilm production (dependent variable) as a function of the categorical variables type of strain (*n* = 8) and type of phenols (*n* = 4) (Figure 4). For each strain, absence of phenols (0 ppm) was used as a control treatment, while average values for tyrosol, hydroxytyrosol, and oleuropein were calculated taking into consideration all their range of concentrations. This global analysis corroborated results previously obtained, which considered the individual concentrations of phenols (Figures 1, 2, 3). Thereby, *L. plantarum* strains Lp15 and LAB23 significantly reduced their biofilm production compared to the control treatment when tyrosol, hydroxytyrosol, or oleuropein were added to the medium (Table 2). *C. boidinii* Y13 clearly was the yeast with the highest biofilm production among yeasts. For none of the yeasts assayed, a statistical difference was noticed when any of the phenolic compounds were added to the culture medium (Table 2). However, results were completely different for the *L. pentosus* strains. The probiotic LPG1 strain considerably increased its biofilm production when tyrosol was added to the medium, which also occurred for the 13B4 strain but in the case of hydroxytyrosol molecule. In the case of Lp119 strain, this microorganism statistically reduced its biofilm production when tyrosol was present in the culture medium (see Table 2).

**TABLE 2:** Factorial ANOVA analysis as a function of type of table olive phenols and strain. Control stands for absence of phenolic compound (0 ppm), while for phenolic compounds, average values were obtained taking into consideration all their individual concentrations.

Microorganism	Strain	Control	Tyrosol	Hydroxytyrosol	Oleuropein
*L. pentosus*	Lp119	1.52 (0.21)^a^	0.76 (0.33)^b^	2.02 (0.82)^a^	1.81 (0.76)^a^
	13B4	1.86 (1.02)^a^	2.37 (1.10)^a^	3.17 (0.74)^b^	2.31 (0.76)^a^
	LPG1	1.88 (0.14)^a^	3.44 (0.75)^b^	2.19 (0.58)^a^	1.92 (0.52)^a^
*L. plantarum*	Lp15	1.53 (0.86)^a^	0.65 (0.36)^b^	0.38 (0.27)^b^	0.38 (0.32)^b^
	LAB23	2.35 (0.46)^a^	1.64 (0.56)^b^	0.88 (0.26)^c^	0.87 (0.37)^c^
Yeasts	Y12	0.35 (0.12)^a^	0.70 (0.45)^a^	0.76 (0.69)^a^	0.75 (0.37)^a^
	Y13	2.83 (0.26)^a^	2.36 (0.44)^a^	2.13 (0.52)^a^	2.18 (0.37)^a^
	Y18	0.67 (0.12)^a^	1.14 (0.66)^a^	0.87 (0.93)^a^	0.53 (0.22)^a^

*Note:* Standard deviation in parentheses. Different superscripts letters, within the same row, stand for significant differences (*p* ≤ 0.05) according to a post hoc Scheffé comparison test.

## 4 Discussion

In this work, we evaluated the influence of three phenolic compounds widely found in table olive fermentations on the biofilm‐forming capacity of various native microorganisms isolated from table olive fermentations. The range of concentrations chosen for tyrosol (0–300 ppm), hydroxytyrosol (0–3000 ppm), and oleuropein (0–3000 ppm) can be easily found during table olive fermentations, albeit polyphenol levels are always closely related to olive cultivar, agronomic traits, ripening level, and processing method (Ruiz‐Barba et al. 2023; Sahan, Cansev, and Gulen 2013).

Diverse in vitro studies have shown the capacity of oleuropein and hydroxytyrosol to significantly reduce biofilm formation in *Staphylococcus* spp. (Crisante et al. 2015; Guo et al. 2023). Phenolic compounds are also considered inhibitors of the LAB growth during table olive fermentations, while they affect the growth of yeasts to a lesser extent (Ruiz‐Barba et al. 1993). Caballero‐Guerrero et al. (2023) found that alpeorujo purified extract rich in hydroxytyrosol (3000 ppm) inhibited the growth of a LAB cocktail formed by diverse *L. pentosus* and *L. plantarum* strains. In this work, we have found that the effects of phenolic compounds on LAB biofilm formation were strain‐dependent. Thereby, an inhibitory effect was observed for the *L. plantarum* strains for the three phenolic compounds assayed, but on the contrary, a stimulating effect was noticed for tyrosol (50 ppm) on the probiotic strain *L. pentosus* LPG1.

*Lactiplantibacillus pentosus* LPG1 was originally isolated from table olive epidermis by Benítez‐Cabello et al. (2019), and it has proved its strong probiotic features as anti‐inflammatory and modulation of the intestinal microbiota agent in diverse animal and clinical studies (Benítez‐Cabello et al. 2020; López‐García, Benítez‐Cabello, Arenas‐de Larriva et al. 2023). During table olive fermentation, LPG1 has the ability to form biofilms on olive epidermis with > 10 million CFU/g between 10 and 30 days of fermentation, which could be related to this ability to increase biofilm formation in the presence of tyrosol (López‐García, Benítez‐Cabello, Tronchoni, et al. 2023). This is an important issue if we wish to turn table olives into a probiotic fermented vegetable. In the same way, we previously reported the presence of genes in the LPG1 strain related to the production of enzymes such as tannase and carboxylesterase, as well as the PadR transcriptional regulator, all of which are involved in the degradation of phenolic compounds (López‐García, Benítez‐Cabello, Ramiro‐García et al. 2023). Specifically, PadR family transcriptional regulators are a group of proteins belonging to the PadR (Phenolic acid decarboxylase Regulator) family, and their main function is to control the expression of genes involved in the degradation or metabolism of phenols and organic acids (Park et al. 2017). Although PadR does not directly transform tyrosol, it can influence the ability of a bacteria to degrade tyrosol. By regulating the expression of genes that encode specific enzymes (such as oxidases or dehydrogenases), PadR may affect the bacteria's ability to process tyrosol if the compound is present in the environment. Carrasco et al. (2018) previously reported the presence of genes involved in tyrosol degradation in *L. pentosus* IG1, a strain isolated from table olives. Moreover, the IG1 strain demonstrated the ability to reduce tyrosol of the medium by 50%, not observed by the *L. plantarum* strains. In our study, we observed a reduction in the biofilm formation by the *L. plantarum* strains in the presence of tyrosol. In contrast, *L. pentosus* LPG1 showed increased biofilm, which could be related to its ability to metabolize this compound. Carrasco et al. (2018) also reported the expression of tannase and esterases in IG1 in the presence of oleuropein. Similarly, tannase and carboxyl esterase genes were found in *L. pentosus* LPG1 in previous studies (López‐García, Benítez‐Cabello, Ramiro‐García et al. 2023). These genes have been described as being involved in the metabolism of oleuropein (Yuan et al. 2015).

On the contrary, olive yeasts were not influenced by the addition of exogenous phenolic compounds in the tested range. Usually tyrosol stimulates the formation of embryonic tubes in yeast cells, further promoting the growth of hyphae and biofilm formation (Rodrigues and Cernáková 2020; Shi et al. 2023). However, Márton, Nagy, and Molnár (2023) concluded that exogenous tyrosol exerted unusual effects on *C. boidinii* growth and biofilm formation ability, depending on temperature, tyrosol concentration, and exposure time.

The microorganisms with the highest ability for in vitro formation of biofilms in the presence of phenols were clearly *L. pentosus* LPG1, *L. pentosus* 13B4, and *C. boidinii* Y13. León‐Romero et al. (2016) reported that among the different pairs of microbial olive strains assayed, the strongest biofilms were obtained from *L. pentosus* and *C. boidinii* co‐cultures. Domínguez‐Manzano (2013) studied the influence of tyrosol, hydroxytyrosol, and oleouropein on the formation of co‐cultures between diverse *L. pentosus* and yeast (*C. bodinii, W. anomalus*, and *S. cerevisiae* ) strains isolated from table olives. In general terms, concentrations of 54 ppm of tyrosol in the medium favored the formation of biofilms in the co‐cultures of *L. pentosus* and *C. boidinii* , while the formation of biofilms in the co‐cultures of *W. anomalus* and *L. pentosus* increased when the concentration of hydroxytyrosol reached 1730 ppm. In the case of oleuropein, the addition of this phenolic compound up to 1730 ppm increased the formation of biofilms in the pairs of *S. cerevisiae* and *L. pentosus* , while for *W. anomalus* no major differences were observed, and in the case of *C. boidinii* , biofilm formation decreased in co‐cultures. All these results confirm those obtained in this work, which showed that biofilm formation by olive microorganisms alone and in co‐cultures in the presence of phenolic compounds is strain‐dependent.

## 5 Conclusion

Further studies are required in order to clarify the metabolic pathways and cellular mechanisms involved in biofilm formation by table olive‐related microorganisms, taking into consideration that the data are strain‐dependent. Results could be very useful to direct the biofilm formation process on the olive epidermis to include probiotic microorganisms such as the *L. pentosus* LPG1 strain, albeit results should be validated with real samples.

## Author Contributions

**Elio López‐García:** data curation (equal), formal analysis (equal), methodology (equal), writing – original draft (equal). **Antonio Benítez‐Cabello:** data curation (equal), methodology (equal), writing – original draft (equal), writing – review and editing (equal). **Francisco Noé Arroyo‐López:** conceptualization (equal), formal analysis (equal), funding acquisition (equal), project administration (equal), supervision (equal), writing – original draft (equal), writing – review and editing (equal).

## Conflicts of Interest

The authors declare no conflicts of interest.

## Data Availability

All data used to prepare this article are included in the tables and figures.
